# Risk factors for post-bronchoscopy pneumonia: a case–control study

**DOI:** 10.1038/s41598-020-76998-z

**Published:** 2020-11-17

**Authors:** Yu Sato, Kengo Murata, Miake Yamamoto, Tsukasa Ishiwata, Miyako Kitazono-Saitoh, Akihiko Wada, Mikio Takamori

**Affiliations:** 1 Department of Respiratory Medicine, Tokyo Metropolitan Tama Medical Center, Tokyo, Japan; 2 Division of Thoracic Surgery, Toronto General Hospital, University Health Network, Toronto, ON Canada; 3 Department of Pulmonology, Tokyo Metropolitan Tama Medical Center, 2-8-29 Musashidai, Fuchu, Tokyo 183-8524 Japan

**Keywords:** Respiratory tract diseases, Bacterial infection

## Abstract

The bronchoscopy, though usually safe, is occasionally associated with complications, such as pneumonia. However, the use of prophylactic antibiotics is not recommended by the guidelines of the British Thoracic Society. Thus far there are few reports of the risk factors for post-bronchoscopy pneumonia; the purpose of this study was to evaluate these risk factors. We retrospectively collected data on patients in whom post-bronchoscopy pneumonia developed from the medical records of 2,265 patients who received 2666 diagnostic bronchoscopies at our institution between April 2006 and November 2011. Twice as many patients were enrolled in the control group as in the pneumonia group. The patients were matched for age and sex. In total, 37 patients (1.4%) had post-bronchoscopy pneumonia. Univariate analysis showed that a significantly larger proportion of patients in the pneumonia group had tracheobronchial stenosis (75.7% vs 18.9%, p < 0.01) and a final diagnosis of primary lung cancer (75.7% vs 43.2%, p < 0.01) than in the control group. The pneumonia group tended to have more patients with a history of smoking (83.8% vs 67.1%, p = 0.06) or bronchoalveolar lavage (BAL) (4.3% vs 14.9%, p = 0.14) than the control group. In multivariate analysis, we found that tracheobronchial stenosis remained an independent risk factor for post-bronchoscopy pneumonia (odds ratio: 7.8, 95%CI: 2.5–24.2). In conclusion, tracheobronchial stenosis was identified as an independent risk factor for post-bronchoscopy pneumonia by multivariate analysis in this age- and sex- matched case control study.

## Introduction

Bronchoscopy is one of the standard methods for diagnosing respiratory diseases including malignant tumors and diffuse lung diseases^1^. Bronchoscopy is usually a safe procedure but is sometimes associated with complications such as pneumonia^2–6^. The incidence of post-bronchoscopy pneumonia ranges from 0.02% to 6.3% and differs according to the reports^2,3,5,6^. Post-bronchoscopy pneumonia is not only a dangerous condition in itself but also delays treatment of the primary disease^2^. A small number of studies have examined the risk factors for post-bronchoscopy pneumonia. Aging, abnormal findings in the bronchial lumen, lung cancer, central location of the tumor, and current smoking have been reported as risk factors for post-bronchoscopy pneumonia, but the reports are inconsistent^2,3,6^. In the current study, we evaluated the risk factors for post-bronchoscopy pneumonia development.

## Materials and methods

Our study was a retrospective, single-center case–control study and was performed in accordance with the Declaration of Helsinki and the guidelines and regulations of the ethics committee of Tokyo Metropolitan Tama Medical Center, which approved the study, including a waiver of informed consent for retrospective data collection and deidentified analysis. All patients 20 years old or older who underwent a diagnostic flexible bronchoscopy for abnormal pulmonary lesions in our bronchoscopy unit were eligible for enrollment. The sampling methods included bronchial washing, bronchoalveolar lavage (BAL), endobronchial brushing, needle aspiration, and forceps biopsy as well as transbronchial needle aspiration (TBNA) with or without endobronchial ultrasound (EBUS) assistance. During the study period, radial EBUS, biopsy via guide sheath, and cryobiopsy were not performed. Patients who received therapeutic bronchoscopy, such as laser resection, argon plasma coagulation, stenting, airway foreign body removal, bronchial occlusion, and balloon dilation, were excluded. Data from the medical records of patients who underwent a bronchoscopy at Tokyo Metropolitan Tama Medical Center, a tertiary teaching hospital in Tokyo, between April 2006 and November 2011, were retrospectively collected, and patients in whom post-bronchoscopy pneumonia developed were extracted for allocation to the pneumonia group.

**Figure 1:**
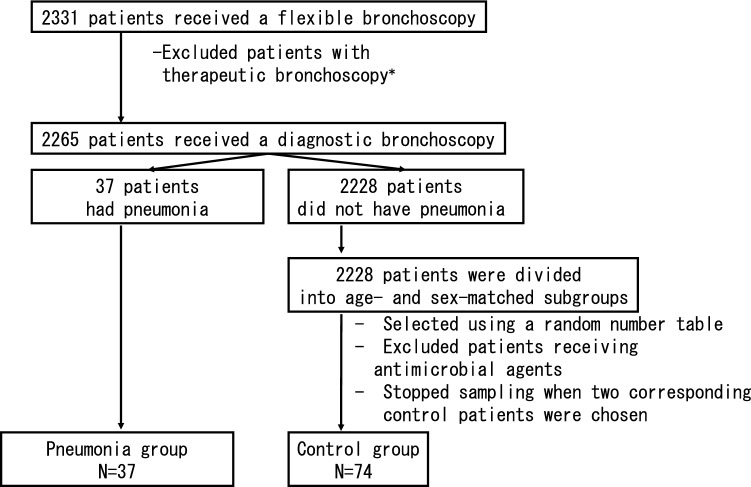
Flowchart of patient selection. Patients without pneumonia were divided into groups by sex and age (by decade). Patients who received antimicrobial agents on the day before their bronchoscopy were excluded from the control group. *Therapeutic bronchoscopy is defined as laser resection, argon plasma coagulation, stenting, airway foreign body removal, bronchial occlusion or balloon dilation.

In the present study, post-bronchoscopy pneumonia was defined as pneumonia or a lung abscess diagnosed by the attending physician, the presence of new pulmonary lesions on a chest radiograph or the development or exacerbation of purulent sputum, and was treated with antibiotics within 30 days after the bronchoscopy. Twice as many patients were randomly selected for the control group as for the pneumonia group and were matched for age and sex, with the latter based on a random number table^7,8^. Patients who received antimicrobial agents were excluded (Fig. 1). Only the initial procedures were included if a patient underwent two or more bronchoscopies.

The factors evaluated were: history of smoking, immunocompromised state, findings of tracheobronchial stenosis, final diagnosis, and bronchoscopic sampling methods. Patients with a history of smoking one year or longer were defined as smokers. An immunocompromised patient was defined as an individual who received corticosteroids, immunosuppressants or chemotherapy or had diabetes, renal failure, liver failure, an hematologic malignancy or asplenia. Tracheobronchial stenosis was defined as the finding of stenosis in any area between the trachea and the subsegmental bronchi by bronchoscopy that was diagnosed by a certified bronchoscopist. The final diagnosis was defined as a diagnosis based on an analysis of the lesions for which bronchoscopy was indicated.

The bronchoscopies were performed under local anesthesia using lidocaine, intramuscular injections of hydroxyzine pamoate or intravenous injections of pethidine without intubation. The bronchoscopes used were 1T200, 1T240, 1T260, F260 (Olympus, Japan). The procedure was performed by two or more experienced respiratory physicians, including one or more bronchoscopists certified by the Japan Society for Respiratory Endoscopy.

The patients’ age was analyzed using Student's t-test while the physician’s experience was analyzed using Mann–Whitney’s U-test. The other univariate analyses were performed using the chi-square test. Multivariate analysis was performed using logistic regression analysis for factors with p < 0.2 on univariate analysis. P < 0.05 indicated statistical significance. SPSS® (version 19) was used for the statistical analysis.

## Results

In 68 months, 2331 patients received 2773 bronchoscopies. Sixty-six patients who received therapeutic bronchoscopies, such as laser resection, argon plasma coagulation, stenting, airway foreign body removal, bronchial occlusion or balloon dilation, were excluded. The median (+ /− SD) age was 65 (+ /− 14) years, and 1393 (61.5%) patients were male. In total, 37 patients (1.4%) had pneumonia. No lung abscess was found. The median duration to pneumonia development after a bronchoscopy was three days (range: 0–20 days). Seventy-four patients who received a bronchoscopy and did not have pneumonia were randomly chosen to be the age- and sex-matched controls (Table 1). Although TBNA with or without EBUS was not excluded, none of the patients in the present study underwent this procedure.

**Table1:** Patient characteristics.

Characteristics	Pneumonia group (N = 37)	Control group (N = 74)
Median age (range), y	67(41–86)	67(47–90)
Male sex	27 (63%)	54 (63%)
**Diagnosis (overlapping)**		
Lung cancer	28	32
Non small cell lung carcinoma	22	27
Squamous cell carcinoma	15	9
Adenocarcinoma	6	17
Small cell carcinoma	6	4
Esophageal cancer	2	0
Non tuberculous mycobacterial infection	2	1
Interstitial pneumonia	1	5
Bronchiectasis	1	1
Bronchial asthma	1	1
Eosinophilic pneumonia	1	1
Mucosa-associated lymphoid tissue lymphoma	1	0
Diffuse large B cell lymphoma	0	1
Metastatic breast cancer	0	1
Tuberculosis	0	3
Sarcoidosis	0	1
Aspiration pneumonia	0	1
Obstructive pneumonia	0	1
Hypersensitivity pneumonitis	0	2
Drug-induced pneumonia	0	1
Mediastinal emphysema	0	1
Bronchopleural fistula	0	1
Inflammatory nodule	0	1
Undiagnosed	0	20
**Bronchoscopic sampling measure (overlapping)**		
Bronchial washing	30	52
Bronchoalveolar lavage	2	11
Insertion of any sampling devices*	32	62
No sampling measure	3	8

*Sampling devices include endobronchial brushes, needles, and forceps.

Univariate analysis showed that the pneumonia group had a significantly larger proportion of patients with tracheobronchial stenosis (75.7% vs 18.9%, p < 0.01) and a final diagnosis of primary lung cancer (75.7% vs 43.2%, p < 0.01) than the control group. The pneumonia group tended to have more patients with a smoking history (83.8% vs 67.1%, p = 0.06) than the control group. With respect to the sampling measures, the pneumonia group had slightly more patients who received BAL than the control group (4.3% vs 14.9%, p = 0.14). There was no significant difference in the physicians’ experience between the two groups (p = 0.26) (Table 2). Of the 28 patients who had tracheobronchial stenosis in the pneumonia group, most (24/28, 86%) had lung cancer. Other diagnoses included esophageal cancer (2/28), nontuberculous mycobacterial infection (1/28), and bronchial asthma (1/28). In contrast, of the 14 patients who had tracheobronchial stenosis in the control group, most (8/14, 57%) also had lung cancer. Other diagnoses included diffuse large B cell lymphoma (1/14), metastatic breast cancer (1/14), obstructive pneumonia (1/14), bronchial asthma, and tuberculosis (1/14). Two patients did not receive a diagnosis.

**Table2:** Univariate analysis of risk factors of post-bronchoscopy pneumonia.

Variables	Pneumonia group (N = 37)	Contorol group (N = 74)	p-value
Tracheobronchial stenosis	28/37 (75.7%)	14/74 (18.9%)	< 0.01
Primary lung cancer	28/37 (75.7%)	32/74 (43.2%)	< 0.01
History of smoking	31/37 (83.8%)	49/73 (67.1%)	0.06
Immunocompromised state	16/37 (43.2%)	24/74 (32.4%)	0.26
Bronchoalveolar lavage	2/37 (4.3%)	11/74 (14.9%)	0.14
Insertion of any sampling devices*	32/37 (86.5%)	62/74 (83.8%)	0.71
Physician's experience (year, median [range])	2 (1–17)	2 (1–17)	0.26

*Sampling devices include endobronchial brushes, needles, and forceps.

Multivariate analysis performed to identify factors with p < 0.20 found that tracheobronchial stenosis was an independent factor in post-bronchoscopy pneumonia (OR: 15.1, 95%CI: 4.8–48.1) (Table 3).

**Table3:** Multivariate analysis of risk factors of post-bronchoscopy pneumonia.

Variables	Odds ratio (95% CI)
Tracheobronchial stenosis	15.1 (4.8–48.1)
Primary lung cancer	2.7 (0.8–9.3.)
History of smoking	3.2 (0.5–19.0)
Bronchoalveolar lavage	1. 9 (0.3–13.1)

## Discussion

Although previous studies identified variables, such as aging, abnormal findings in the bronchial lumen, lung cancer, central location of the tumor, and current smoking as risk factors^2,3,5,6^ (Table 4), this age- and sex-matched controlled study demonstrated that tracheobronchial stenosis was the only independent risk factor for post-bronchoscopy pneumonia.

**Table 4:** Comparison of studies evaluating risk factors of post-bronchoscopy pneumonia.

Study	Study design	Number of patients	Post bronchoscopy pneumonia incidence	Risk factors of post bronchoscopy pneumonia	Factors unrelated to the risk of post-bronchoscopy pneumonia
Pereira et al.^6^	Prospective	100	6%	Aging	Lung cancer
				Abnormal bronchoscopic findings	Duration of bronchoscopy
					Lidocaine consumption
					Bronchoscopic sampling method
					Cough
					Sputum
					Oral hygiene prophylaxis with antibiotics
					Smoking history
Kanemoto et al.^3^	Prospective	358	5.6%	ND	Aging
Kanazawa et al.^5^	Prospective	931	1.6%	Abnormal bronchoscopic findings	ND
				Bronchial carcinoma	
Takiguchi et al.^2^	Retrospective	237	6.3%	Aging (age≧70 yrs)	ND
				Current smoking	
				Central location of tumor	
Shimoda et al.^13^	Retrospective	327	6.1%	Necrosis and/or cavity in tumor	Aging
				Large tumor diameter	Current smoking status
				Low serum albumin level (< 4.0 g/dL)	Abnormal bronchoscopic findings
Our study	Retrospective	2265 (Penumonia: 37, Control: 74)	1.4%	Tracheobronchial stenosis	Immunocompromised status
					Primary lung cancer
					History of smoking
					Bronchoalveolar lavage
					Insertion of any device
					Physician's experience

ND, not detected.

The mechanism of post-bronchoscopy pneumonia in patients with tracheobronchial stenosis can be adequately explained based on that of obstructive pneumonia: first, intramural or extramural stenosis of the normal bronchi occurs due to primary lung cancer, metastatic lung tumor, benign tumor, hematoma, edema, foreign body, pleural effusion or fibrosis. Second, progression to endogenous lipoid pneumonia occurs due to the accumulation of foamy macrophages and mucus and cell invasion caused by disorders of the mucociliary transport system distal to the stenosis. Finally, development of obstructive pneumonia occurs via colonization of bacteria flowing into the lesion^9,10^. Bronchoscopy can aggravate this condition by carrying the oropharyngeal flora attached to the bronchoscope into the lesion, thereby leading to obstructive pneumonia.

Although several previous studies suggested that lung cancer was associated with post-bronchoscopy pneumonia based on univariate analysis^2,3^ (Table 4), lung cancer itself was not identified as an independent risk factor on multivariate analysis in our study. Pneumonia developed in four patients in the pneumonia group with bronchial stenosis who did not have lung cancer while pneumonia did not develop in any of the patients in the control group with lung cancer. Indeed, some reports showed that a predisposition for pneumonia was caused by tracheobronchial stenosis due to diseases other than lung cancer. Pneumonia developed in 4.2% of the patients with endobronchial valves for advanced emphysema, and most of the cases were resolved by antibiotic treatment and valve removal^11^. Obstructive pneumonia also developed due to occlusion by endobronchial Watanabe spigots for refractory pneumothorax and resolved through antibiotic treatment and spigot removal^12^. A recent study suggested that the central tumor location was one of the risk factors for post-bronchoscopy pneumonia. However, the study did not analyze the bronchoscopic findings and only included patients with lung cancer, which was difficult to diagnose during the bronchoscopy^2^. Another study suggested that patients with lung cancer with necrosis and/or cavity or large tumor diameter tend to develop a lung abscess^13^. In the article, the authors suggested that radial EBUS using the guide-sheath (EBUS-GS) technique might contribute to the incidence of lung abscess in patients with lung cancer. However, patients with non-neoplastic lung diseases who do not receive EBUS-GS also acquired pneumonia after bronchoscopy. While the previous study only included patients with lung cancer, the present study included patients with any pulmonary lesions. Notably, there were no patients with a lung abscess in our study. This clear distinction might be due to differences in the patients’ background, sampling methods or the developmental mechanisms of post-bronchoscopy respiratory infection. Thus, our study suggested that it was not lung cancer itself, but the bronchial stenosis caused by any of a number of diseases (including lung cancer), that directly induced post-bronchoscopy pneumonia.

Multivariate analysis did not demonstrate that smoking history was a risk factor for post-bronchoscopy pneumonia despite the promising results of the univariate analysis. Smoking, which is well-known as a risk factor for numerous lung diseases, such as lung cancer, chronic bronchitis, and chronic obstructive pulmonary diseases, can also disturb mucociliary transportation, increase sputum production, and depress the cough reflex^14,15^. Indeed, a retrospective study of 237 patients with lung cancer who underwent a bronchoscopy reported that current smoking was a risk factor^2^. However, a previous prospective study of 100 patients who underwent a bronchoscopy suggested that smoking history was not associated with post-bronchoscopy pulmonary complications including pneumonia^6^, and a study that compared obstructive pneumonia and bacterial, community-acquired pneumonia suggested that smoking was not a risk factor for obstructive pneumonia^9^. While smoking might directly or indirectly exacerbate post-bronchoscopy pneumonia, its exact role is still unknown.

There are no widely accepted criteria for diagnosing post-bronchoscopy pneumonia, and the definition and observation period for this condition vary^2,3,5,6^. We defined pneumonia occurring within 30 days after the procedure as a bronchoscopy-related infection in accordance with the surgical-site infection guidelines issued by the Centers for Disease Control and Prevention, which state that an organ infection occurring within 30 days after an operation is likely to be related to the operation if no implant has been left in place^16^. Our study did not require radiographic confirmation because chest X-rays may fail to detect pneumonia^17^.

Our study concluded that tracheobronchial stenosis is the only independent risk factor for post-bronchoscopy pneumonia. Tracheobronchial stenosis can be judged promptly during bronchoscopy, and there is no need to wait for the diagnosis. Also, whether tracheobronchial stenosis is caused by lung cancer or not is irrelevant. Our study enrolled the largest number of patients to date for the purpose of determining the risk factors for post-bronchoscopy pneumonia. However, it has some limitations. First, since the study was retrospective and a definitive diagnosis of post-bronchoscopy pneumonia is difficult to establish, the condition might have been underdiagnosed. However, since our criteria for pneumonia included antibiotic administration, few cases of pneumonia were likely to have been overlooked. Second, immune status and bronchoscopic sampling methods varied. A carefully stratified evaluation is needed to address these issues. Third, the role of age and sex was not able to be examined because our study was designed as an age- and sex-matched control study. Finally, since patients who received EBUS-GS, endobronchial cryobiopsy or therapeutic bronchoscopy were not included, the issue of whether tracheobronchial stenosis contributed to post-bronchoscopy pneumonia development in these patients was unable to be addressed.

## Conclusion

The present case–control study matching patient age and sex was the first to demonstrate by multivariate analysis that stenosis of the trachea or bronchi is an independent risk factor for the development of post-bronchoscopy pneumonia. This finding will help to identify patients at risk of post-bronchoscopy pneumonia development without the need to wait for a pathological diagnosis.
